# Development of a Sacha Inchi Oil-Based Nanoemulsion Containing Mangosteen Pericarp Extract and Its Antioxidant Activity

**DOI:** 10.3390/pharmaceutics18091072

**Published:** 2026-08-27

**Authors:** Nur Aisyah, Ikra Nurohman, Sriwidodo Sriwidodo, Patihul Husni, Cecep Suhandi, Gofarana Wilar, Ahmad Choibar Tridakusumah, Sabreena Safuan

**Affiliations:** 1 Department of Pharmaceutics and Pharmaceutical Technology, Faculty of Pharmacy, Universitas Padjadjaran, Sumedang 45363, Indonesia; nur23066@mail.unpad.ac.id (N.A.); ikra24001@mail.unpad.ac.id (I.N.); patihul.husni@unpad.ac.id (P.H.); 2 Center of Pharmaceutical Formulation Development, Faculty of Pharmacy, Universitas Padjadjaran, Sumedang 45363, Indonesia; cecep17001@mail.unpad.ac.id; 3 Department of Pharmacology and Clinical Pharmacy, Faculty of Pharmacy, Universitas Padjadjaran, Sumedang 45363, Indonesia; g.wilar@unpad.ac.id; 4 Department of Agricultural, Faculty of Agriculture, Universitas Padjadjaran, Sumedang 45363, Indonesia; ahmad.choibar@unpad.ac.id; 5 Department of Biomedicine, School of Health Sciences, Universiti Sains Malaysia, Kubang Kerian 16150, Kelantan, Malaysia; sabreena@usm.my

**Keywords:** mangosteen pericarp extract, sacha inchi oil, nanoemulsion, antioxidant

## Abstract

**Background/Objectives:** Mangosteen pericarp extract (*Garcinia mangostana* L.) exhibits robust antioxidant properties. However, its pharmaceutical application is hindered by poor water solubility and low physicochemical stability. This study aimed to develop a lipid-based nanoemulsion to overcome these limitations and assess its physicochemical characteristics, radical scavenging capacity, and dissolution profiles. **Methods:** Nanoemulsions were prepared by high-shear homogenization followed by ultrasonication method using sacha inchi oil (*Plukenetia volubilis* L.) across varying hydrophilic–lipophilic balance (HLB) values. Formulations were characterized by emulsion type, pH, rheology, droplet size, surface charge, encapsulation efficiency, and morphology via transmission electron microscopy. Antioxidant capacity was quantified using the DPPH assay, while in vitro dissolution experiments measured solubility enhancement relative to unformulated extract. **Results:** All formulations formed stable oil-in-water systems without phase separation. The HLB 10 formulation exhibited optimal performance, yielding a mean droplet diameter of 490.03 ± 32.12 nm, zeta potential of −52.73 ± 0.09 mV, and encapsulation efficiency of 89.14 ± 0.19%. Micrographs revealed well-structured spherical droplets. The raw extract showed strong antioxidant activity (IC_50_ = 21.68 ppm), which remained functional in nanoemulsion (IC_50_ = 84.48 ppm). Dissolution testing indicated a 13.4-fold improvement over raw powder. However, storage evaluation indicated chemical loss of bioactive constituents over one month. **Conclusions:** Sacha inchi oil-based nanoemulsions significantly enhance the dissolution rate of mangosteen pericarp extract while maintaining functional antioxidant potential. Although chemical stability during storage remains a limitation requiring further optimization, this nanocarrier platform offers strong potential to overcome solubility barriers for oral bioactive delivery.

## 1. Introduction

Mangosteen (*Garcinia mangostana* L.) is a tropical fruit widely distributed in Southeast Asia. Its pericarp contains various bioactive compounds, particularly xanthones such as α-mangostin, which exhibit strong antioxidant activity [1]. However, the application of mangosteen pericarp extract in pharmaceutical formulations is limited by the poor aqueous solubility and physicochemical instability of its major bioactive compounds [2,3]. These limitations may reduce dissolution and consequently restrict the effective delivery of α-mangostin [3,4]. Consequently, formulating state-of-the-art nano-delivery configurations has become an important approach to optimize the bioavailability of lipophilic phytochemical compounds [5].

Lipid-based delivery systems have been widely investigated to improve the dissolution and delivery of poorly water-soluble bioactive compounds [6,7]. Among these systems, submicron emulsions are attractive because they can incorporate lipophilic compounds into an oil phase and disperse them in an aqueous continuous phase, the formation of small droplets can provide a large interfacial area, which may facilitate interaction with the aqueous medium and improve the apparent dissolution of incorporated compounds [8,9]. The performance of such systems depends on several formulation factors, particularly the type and concentration of oil and surfactant and the hydrophilic–lipophilic balance (HLB) of the surfactant system.

Previous studies have investigated lipid-based delivery systems for improving the dissolution and stability of poorly water-soluble plant-derived compounds [8,10]. Several studies have also explored lipid-based formulations of mangosteen extract or α-mangostin using different lipid phases and surfactant systems. These studies demonstrate the potential of lipid-based formulations to improve the dispersion and dissolution of mangosteen-derived bioactive compounds. Sacha inchi oil was selected as the oil phase because of its distinctive fatty acid composition, particularly its high content of polyunsaturated fatty acids, including omega-3 and omega-6, which may provide a functional advantage as a plant-derived lipid carrier for lipophilic bioactive compounds [11,12]. However, the use of sacha inchi oil (*Plukenetia volubilis* L.) as the oil phase in a submicron emulsion containing mangosteen pericarp extract has been insufficiently investigated [13,14,15].

Therefore, the novelty of this study lies in the use of sacha inchi oil as a plant-derived lipid carrier for mangosteen pericarp extract and the comprehensive evaluation of the resulting submicron emulsion. We hypothesized that incorporating mangosteen pericarp extract into a sacha inchi oil-based submicron emulsion would produce a system with acceptable physicochemical characteristics and improve the dissolution and antioxidant performance of the extract compared with the non-formulated extract. Accordingly, this study aimed to develop a sacha inchi oil-based submicron emulsion containing mangosteen pericarp extract using high-shear homogenization followed by ultrasonication and to evaluate its physicochemical characteristics, antioxidant activity, and in vitro dissolution behavior.

## 2. Materials and Methods

### 2.1. Raw Materials and Reagents

Mangosteen pericarp extract was supplied by Labcos (Bandung, Indonesia), and sacha inchi oil was supplied by PT Quilla Herbal Indonesia Sejahtera (Bandung, Indonesia). The mangosteen pericarp extract was used as received and contained 36.71 ± 0.70% α-mangostin, as determined by TLC and HPLC. Sacha inchi oil was obtained from *Plukenetia volubilis* L. seeds sourced from Cianjur, West Java, Indonesia, and had a relative density of 0.91 g/cm^3^, an acid value of 0.38 ± 0.02 mg/g, a peroxide value of 11.01 mEq/kg, and fatty acid contents of 48.50% omega-3, 34.80% omega-6, and 7.70% omega-9. Tween 80, Span 80, xanthan gum, sodium benzoate, and butylated hydroxytoluene (BHT) were purchased from Mitra Chem (Bandung, Indonesia). The α-mangostin reference standard was obtained from MerkHerb (Bandung, Indonesia), while methanol, ethanol, *n*-hexane, and ethyl acetate were purchased from Merck (Darmstadt, Germany). DPPH was obtained from Sigma-Aldrich (St. Louis, MO, USA). Deionized ultrapure water was used throughout the experiments.

### 2.2. Qualitative Identification of α-Mangostin

Thin-layer chromatography (TLC) was utilized for the preliminary verification of α-mangostin within the mangosteen pericarp extract. Samples and α-mangostin standard solutions were spotted onto silica gel GF254 plates and developed using an ethyl acetate to n-hexane mixture (1:1, *v*/*v*) as the mobile phase. Following development, the chromatographic plates underwent UV visualization at a wavelength of 254 nm to calculate their respective retardation factors (Rf) [16].

### 2.3. Quantitative Determination of α-Mangostin by HPLC

To quantify the α-mangostin content, a high-performance liquid chromatography (HPLC) system equipped with a photodiode array detector (Alliance Waters 2695/2996, Waters Corp., Milford, MA, USA) was employed. Elution was performed on a C18 column (150 × 4.6 mm, 5 μm), utilizing a mobile phase blend of methanol and water (85:15, *v*/*v*). The operation was maintained at a flow rate of 1.0 mL/min and an injection volume of 10 μL, with spectrophotometric monitoring set at 318 nm. A series of standard solutions of α-mangostin (20−100 μg/mL) were generated to establish the standard calibration curve. For the analysis, the extract samples were first solubilized in methanol, passed through a 0.45 μm syringe membrane filter, and subsequently run under the same chromatographic parameters [17].

### 2.4. Formulation of Mangosteen Pericarp Extract Nanoemulsions

The fabrication of nanoemulsions involved a high-shear emulsification approach, which was subsequently subjected to probe ultrasonication [18,19]. Eight formulations with different hydrophilic–lipophilic balance (HLB) parameters spanning from 8 up to 15 were developed by varying the blending ratios of Tween 80 and Span 80 while keeping the cumulative surfactant loading fixed at 10% *w*/*w*. The detailed constituent setup for individual batches is outlined in Table 1.

The water phase comprised Tween 80 (hydrophilic surfactant), sodium benzoate (preservative), xanthan gum (thickening and viscosity-enhancing agent), and purified water (vehicle). Blending of these components was carried out via a high-speed homogenizer operating at 20,000 rpm for a 3-min duration under ambient temperature. In a separate vessel, preparation of the lipid phase involved dissolving the active ingredient (mangosteen pericarp extract) and the antioxidant stabilizer (BHT) in sacha inchi oil pre-mixed with Span 80 (lipophilic surfactant). To form a crude dispersion, the lipid phase was introduced dropwise into the water phase while maintaining continuous homogenization. Following this, the resulting macroemulsion underwent probe ultrasonication (70% amplitude, 0.5 s pulse cycle) for 30 min to yield the final nanoemulsion system.

### 2.5. Physicochemical Characterization

#### 2.5.1. Organoleptic Assessment and Emulsion Classification

Macroscopic inspection of the nanoemulsions was conducted to monitor color, uniformity, transparency, and potential phase separation. To identify the emulsion type, the dye solubility method using methylene blue was executed. A homogeneous dispersion of the water-soluble dye served as confirmation of an oil-in-water (O/W) nanoemulsion architecture [20].

#### 2.5.2. Particle Size, Polydispersity Index, and Zeta Potential

A SZ-100 Nanoparticle Size and Zeta Potential Analyzer (HORIBA, Kyoto, Japan) utilizing dynamic light scattering (DLS) was employed to measure the average droplet size, polydispersity index (PDI), and zeta potential of the formulations. To mitigate multiple scattering phenomena, a 10-fold dilution of each sample with deionized ultrapure water was performed ahead of analysis. All data points were recorded in triplicate at a controlled temperature of 25 °C [8].

#### 2.5.3. Encapsulation Efficiency

The quantification of encapsulation efficiency (EE) relied on isolating unencapsulated α-mangostin from the nanoemulsion droplets via centrifugal ultrafiltration. To ensure effective separation of free bioactive molecules while preventing artificial precipitation of undissolved extract particles, moderate centrifugation parameters (12,000 rpm for 20 min) were applied following the validated protocol described by Nan et al. [21]. The supernatant was subsequently filtered through a 0.45 μm membrane. HPLC analysis under the aforementioned parameters was then used to determine the concentration of free α-mangostin remaining within the supernatant phase.

The calculation for entrapment efficiency was executed using the equation below:

$$EE\left(\%\right)=\frac{W_{t}-W_{f}}{W_{t}}\times 100$$

in which W_t_ represents the cumulative quantity of α-mangostin introduced into the system, whereas W_f_ denotes the free portion of α-mangostin quantified from the supernatant phase.

#### 2.5.4. Determination of pH

An analytical pH meter (Mettler Toledo Seven Easy S20, Greifensee, Switzerland), previously calibrated, was utilized to record the pH values of the nanoemulsions at ambient temperature. These assays were run in triplicate [22].

#### 2.5.5. Determination of Rheological Properties

The evaluation of rheological behavior was performed with a rotational viscometer utilizing spindle No. 2 at a rotational speed of 60 rpm under room temperature conditions. Every measurement was conducted in triplicate [22].

### 2.6. Morphological Characterization

Transmission electron microscopy (TEM; HT7700, Hitachi, Tokyo, Japan) was utilized to observe the structural appearance of the optimized nanoemulsion. To prepare for imaging, a single droplet of the diluted sample was placed onto a copper grid and left to dry at ambient temperature. The resulting micrographs were captured at suitable magnification levels to assess the structural features and droplet morphology [8].

### 2.7. In Vitro Release Study

To examine the release patterns of the mangosteen pericarp extract incorporated within the nanoemulsion, a dialysis bag diffusion technique was implemented [10]. In short, a 5 mL volume of the nanoemulsion was transferred into a dialysis membrane bag, which was then submerged inside a phosphate-buffered solution (pH 6.8) maintained at a constant temperature of 37 ± 0.5 °C while being stirred continuously at 50 rpm. Unformulated mangosteen extract and pure sacha inchi oil served as control benchmarks.

Sampling was executed at specific time points (0, 5, 10, 20, 30, 60, 120, 180, 240, 300, and 360 min), during which fixed aliquots were extracted from the dissolution environment and immediately replenished with an identical volume of pristine, pre-warmed buffer medium. The amount of dissolved α-mangostin was subsequently measured via spectrophotometric analysis to determine the cumulative percentage of active compound release.

### 2.8. Radical Scavenging Capacity Assay

The DPPH radical scavenging assay was employed to evaluate the antioxidant capacity of the mangosteen pericarp extract, pure sacha inchi oil, and the optimized nanoemulsion system [8]. Dilutions of the samples were prepared using ethanol to achieve a range of concentrations. A 1 mL portion of the sample solution was combined with an equal volume (1 mL) of DPPH radical solution (0.2 mM), followed by a 30-min incubation period in a dark environment at room temperature.

A UV-Vis spectrophotometer (UNICO 1200, Dayton, NJ, USA) was used to record the optical density at 515 nm. The percentage of radical scavenging effect was computed using the expression below:

$$Inhibition\left(\%\right)=\frac{A_{c}-A_{s}}{A_{c}}\times 100$$

in which A_c_ denotes the optical density of the control group, whereas A_s_ represents the optical density of the tested sample. Concentration-response curves were then constructed to calculate the IC_50_ values.

### 2.9. Stability Assessment

To evaluate its robustness, the optimized nanoemulsion was exposed to stability testing across a pair of distinct thermal environments: 25 ± 2 °C and 40 ± 2 °C. The formulations were maintained under these settings for a one-month duration, with comprehensive tracking performed on the initial and final days of the evaluation window. The monitored indicators comprised organoleptic properties, droplet dimensions, PDI, surface charge, pH, rheological behavior, quantification of α-mangostin, along with radical scavenging efficacy. Any modifications regarding the system’s physicochemical attributes throughout the storage timeframe were thoroughly documented and scrutinized [23].

### 2.10. Statistical Analysis

Every experiment was run in triplicate, with data points presented as the mean value ± standard deviation (SD). Computation of statistical models was executed utilizing the R software environment version 4.4.1 (R Foundation for Statistical Computing, Vienna, Austria) [9]. Data distribution behavior was verified through the Shapiro–Wilk normality test, whereas the equality of variances was examined using Levene’s test. In cases where datasets conformed to both variance homogeneity and normal distribution criteria, variance variations among individual batches were parsed via one-way analysis of variance (ANOVA) combined with Tukey’s post hoc test. Conversely, for datasets violating the assumptions of normality, non-parametric comparisons were performed utilizing the Kruskal–Wallis method paired with Dunn’s multiple comparison test, applying a Bonferroni adjustment.

To validate the choice of the optimized batch, the designated formulation underwent comparative evaluation against the combined aggregate of all other batches. Depending on how the underlying data was distributed, these two-group evaluations relied on either Welch’s *t*-test or the non-parametric Wilcoxon rank-sum test. The threshold for determining statistical significance was set at a *p*-value < 0.05.

## 3. Results and Discussion

### 3.1. Qualitative and Quantitative Determination of α-Mangostin

#### 3.1.1. Qualitative Identification of α-Mangostin

**Figure 1:**
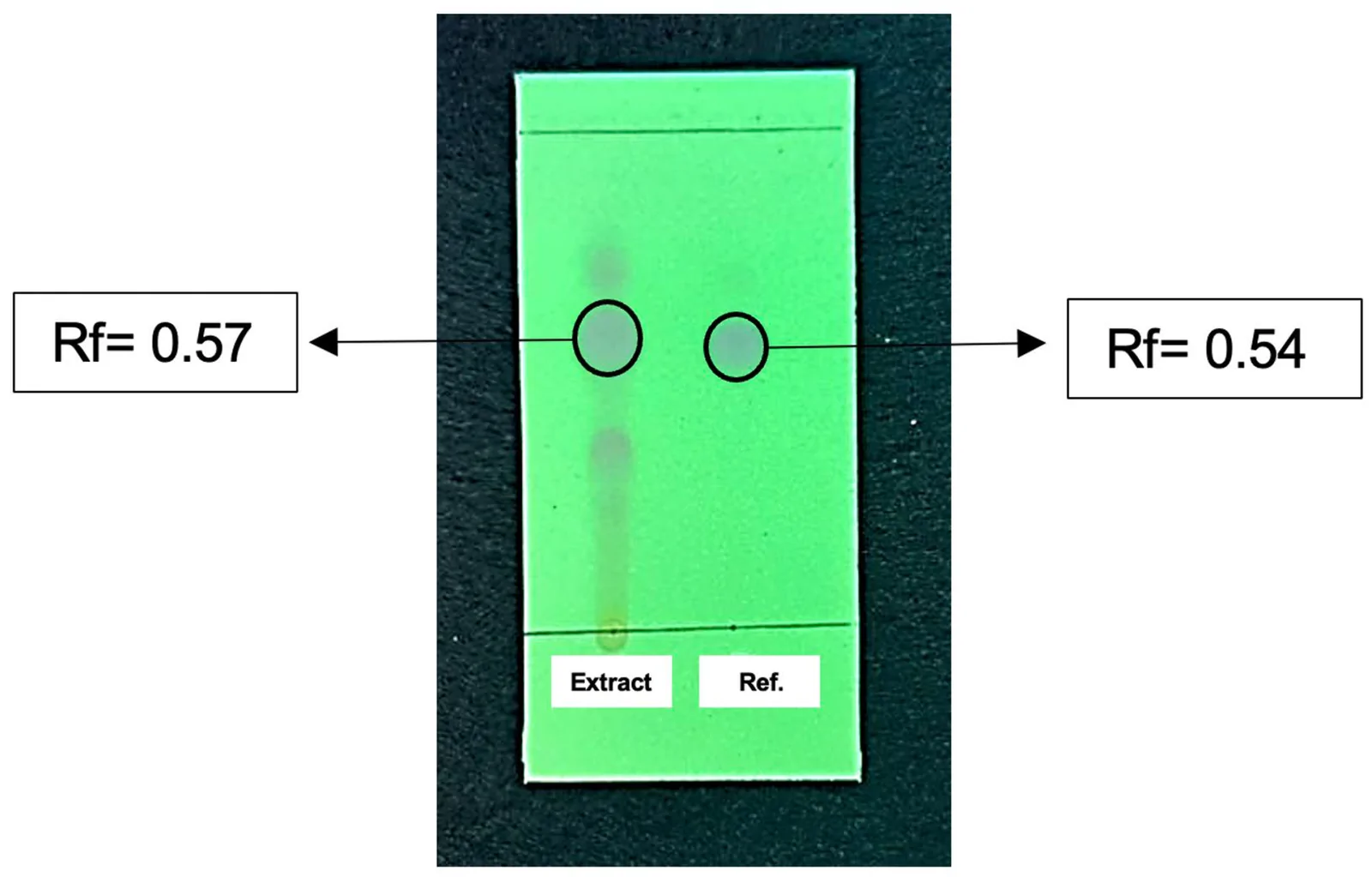
TLC chromatograms illustrating the detection of α-mangostin within the pericarp extract of mangosteen. Thin-layer chromatograms of α-mangostin standard and mangosteen pericarp extract developed using silica gel GF254 plates (Merck, Darmstadt, Germany).

Thin-layer chromatography (TLC) served as the primary tool to confirm the presence of α-mangostin in the mangosteen pericarp extract. As illustrated in Figure 1, the tested sample generated a distinct chromatographic spot exhibiting an R_f_ value of 0.57, which closely aligned with the spot produced by the authenticated reference standard of α-mangostin (R_f_ = 0.54). This minor deviation observed in the retardation factors (ΔR_f_ = 0.03) substantiated the successful identification of α-mangostin within the raw plant extract.

#### 3.1.2. Quantitative Determination of α-Mangostin

**Figure 2:**
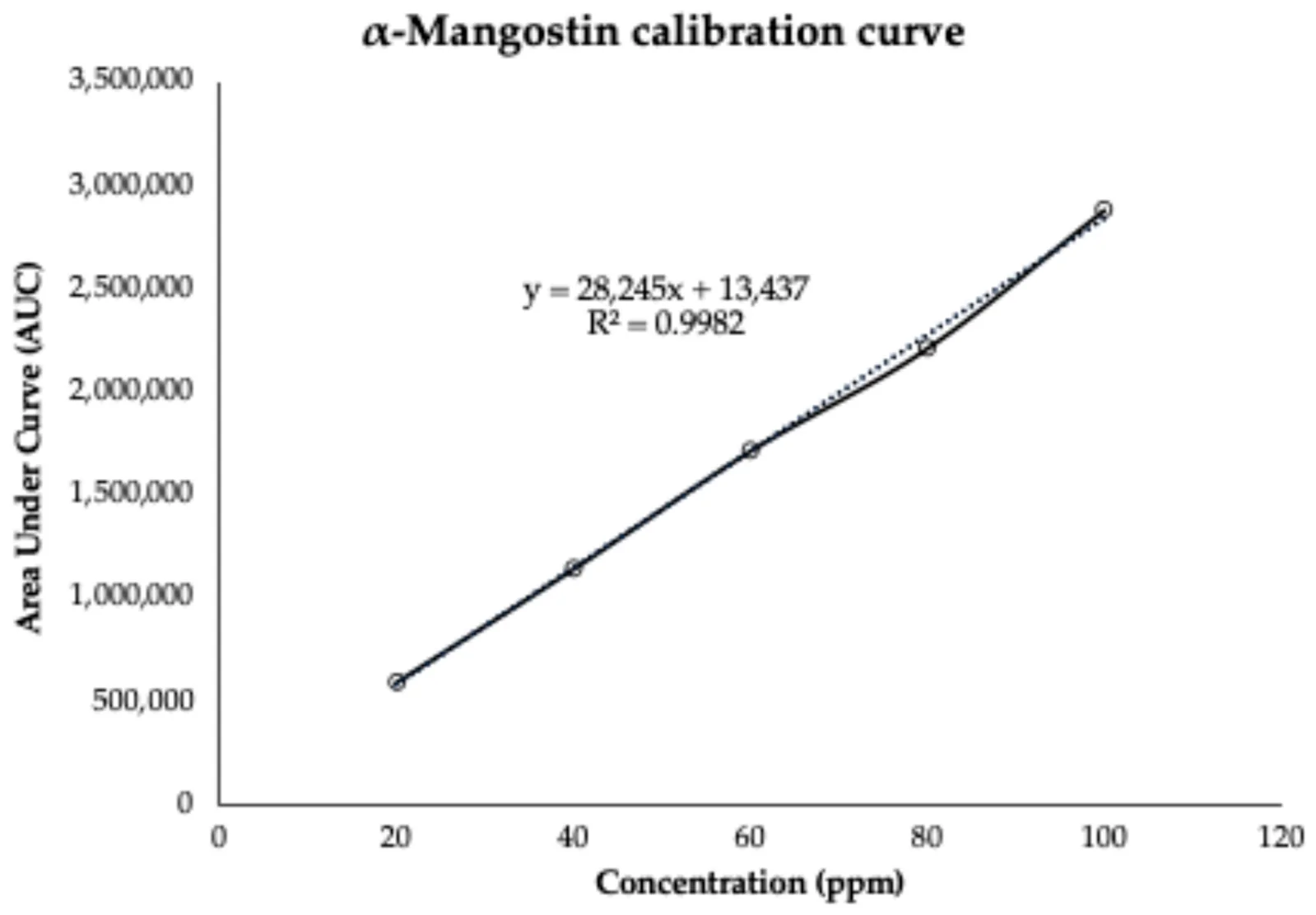
Calibration curve of α-mangostin standard determined by HPLC.

Quantitative determination of α-mangostin was performed using High-Performance Liquid Chromatography (HPLC). Standard solutions of α-mangostin at various concentrations (20–100 μg/mL) were prepared to construct a calibration curve by plotting peak area against standard concentration. Subsequently, linear regression analysis was conducted to obtain the calibration equation and the coefficient of determination (R^2^), as shown in Figure 2. The α-mangostin content in the sample was then calculated based on the obtained calibration equation. Chromatographic analysis revealed a distinct peak corresponding to α-mangostin at a retention time of 7.63 min. Based on the quantification results, the total α-mangostin content in the mangosteen pericarp extract was 36.71 ± 0.70%, confirming that the extract contains a significant amount of the target xanthone compound.

### 3.2. Initial Screening of Nanoemulsified Batches

Various nanoemulsion combinations were fabricated by blending Tween 80 and Span 80 at specific ratios to yield HLB parameters spanning from 8 to 15. Preliminary stability screening was conducted through visual observation during one month of storage. Out of all tested batches, those with HLB values of 8, 9, 10, 14, and 15 demonstrated excellent physical integrity, showing no signs of macroscopic phase separation. In contrast, systems prepared under HLB conditions of 11, 12, and 13 developed visible instability (creaming and phase separation) during the storage period. This localized instability at intermediate HLB values can be attributed to an optimal surfactant packing parameter shift or an intermediate surfactant curvature that fails to sufficiently reduce interfacial tension or provide adequate steric repulsion compared to the extremes of the HLB spectrum [24,25]. Consequently, batches with HLB 11, 12, and 13 were excluded from subsequent characterization, and the physically stable formulations were selected for further physicochemical evaluation.

### 3.3. Physicochemical Properties of Nanoemulsions

#### 3.3.1. Organoleptic Evaluation and Emulsion Classification

**Figure 3:**
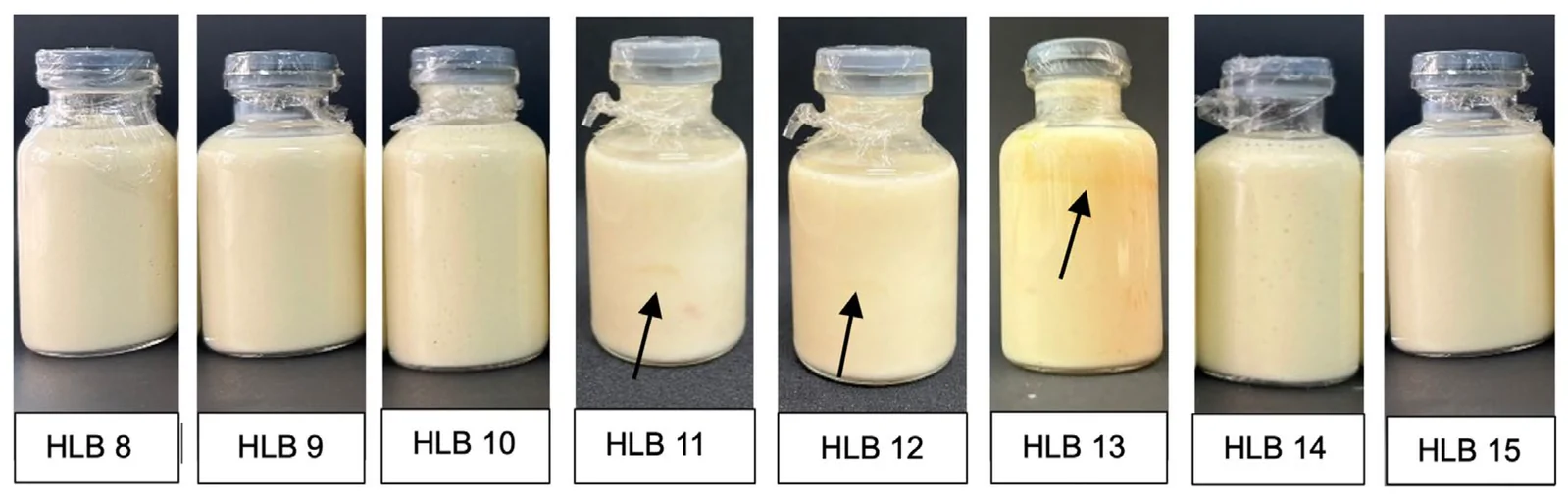
Visual appearance of mangosteen pericarp extract nanoemulsions prepared at different HLB values. The black arrow indicates physical changes leading to instability, manifested as phase separation.

The selected formulations (HLB 8, 9, 10, 14, and 15) appeared as homogeneous yellowish dispersions with no visible sedimentation or phase separation (Figure 3). All formulations exhibited a smooth texture and retained the characteristic odor of sacha inchi oil throughout the observation period.

Dye solubility testing using methylene blue confirmed that all formulations formed an oil-in-water (O/W) nanoemulsion architecture. Uniform distribution of the hydrophilic dye throughout the formulation indicated that water constituted the continuous phase.

#### 3.3.2. Particle Size, Polydispersity Index, and Zeta Potential

Data obtained from dynamic light scattering (DLS) highlighted that the surfactant blend’s HLB parameters exerted a clear influence over the physicochemical characteristics of the nanoemulsions (Table 2). The mean droplet size ranged from 490.03 ± 32.12 nm to 987.27 ± 67.49 nm. Among all formulations, HLB 10 produced the smallest droplet diameter.

Statistical analysis confirmed a significant reduction in particle size for HLB 10 compared with the remaining formulations (490.03 ± 32.12 nm vs. 861.84 ± 167.31 nm, Wilcoxon test, *p* = 0.012). This result demonstrates that the surfactant combination corresponding to HLB 10 most effectively reduced interfacial tension and promoted droplet formation.

The recorded PDI values spanned between 0.65 ± 0.04 and 0.89 ± 0.03. Although HLB 10 exhibited a lower PDI than HLB 8 and HLB 9, the difference was not statistically significant when compared with the pooled non-optimal formulations (*p* > 0.05), indicating comparable size homogeneity among the investigated nanoemulsions.

The zeta potential values ranged from −52.73 ± 0.09 mV to −60.70 ± 1.13 mV. Statistical analysis revealed a significant difference between HLB 10 and the remaining formulations (*p* = 0.036). Nevertheless, all formulations exhibited zeta potential magnitudes greater than 30 mV, indicating sufficient electrostatic stabilization and good colloidal stability.

The successful development of these colloidal systems depended heavily on the HLB parameters of the surfactant mixture. Among the formulations evaluated, HLB 10 produced the most optimal physicochemical attributes, notably yielding the minimum droplet diameter, alongside satisfactory colloidal stability. The observed behavior indicates that the selected Tween 80–Span 80 ratio provided an optimal reduction of boundary tension across the oil-water interface, facilitating efficient droplet disruption during homogenization and preventing extensive recoalescence [21,26]. Similar observations have been reported in vegetable oil-based nanoemulsion systems, where matching the required HLB of the oil phase significantly improves emulsification efficiency and long-term stability [27,28].

Taking into account the collective data regarding droplet diameter, PDI, and dispersion stability, HLB 10 was chosen as the ideal system, which was then directed to further characterization. To further assess the influence of extract incorporation on nanoemulsion properties, the optimized nanoemulsion containing mangosteen extract was compared with a blank nanoemulsion prepared under identical conditions (Table 3).

The incorporation of mangosteen pericarp extract increased the mean droplet size from 191.03 ± 2.76 nm to 490.03 ± 32.12 nm. Likewise, the PDI increased from 0.35 ± 0.01 to 0.66 ± 0.01, whereas the magnitude of the negative zeta potential increased from −42.50 ± 0.26 mV to −52.73 ± 0.09 mV. Although the mean droplet size of the optimized formulation approached the upper boundary of the sub-micron scale (490.03 ± 32.12 nm), it remains well within the scientifically accepted range for nanoemulsions, which is broadly defined as lipid droplets between 20 and 500 nm [29,30]. Furthermore, while a PDI < 0.3 typically signifies a highly monodisperse population in single-component synthetic formulations, a PDI below 0.7 is considered acceptable for complex lipid-based carrier systems incorporating crude botanical extracts, indicating a sufficiently uniform size distribution without severe aggregation [31].

Although the particle size was larger than that reported for some previously developed mangosteen nanoemulsion systems, the formulation remained within the submicron/nanoscale range used for colloidal drug delivery. For comparison, Krisanti et al. reported a particle size of approximately 353 nm for a mangosteen pericarp extract nanoemulsion prepared using a low-energy emulsification approach. The difference in particle size may be related to differences in oil phase composition, surfactant ratio, extract loading, and emulsification method [30]. The highly negative zeta potential observed in the present study indicates strong electrostatic repulsive forces among the dispersed droplets, thereby minimizing particle clustering and promoting superior dispersion integrity [32,33]. The presence of xanthan gum may also have contributed to the negative surface charge by introducing anionic functional groups at the droplet interface [34]. In addition, the relatively narrow particle size distribution obtained for the optimized formulation suggests adequate homogeneity of the dispersed system.

#### 3.3.3. Encapsulation Efficiency

The encapsulation efficiency (EE) of mangosteen pericarp extract in the nanoemulsion formulations is presented in Table 2. All formulations exhibited high encapsulation performance, with EE values ranging from 84.76 ± 4.48% to 89.14 ± 0.19%. Although HLB 10 exhibited the highest mean EE value, statistical analysis indicated no significant difference in encapsulation efficiency between HLB 10 and the remaining formulations (*p* > 0.05). These findings suggest that efficient incorporation of mangosteen pericarp extract into the sacha inchi oil phase was achieved across a relatively broad HLB range. Consequently, the selection of HLB 10 as the optimized formulation was primarily driven by its significantly smaller particle size while maintaining high encapsulation efficiency and satisfactory colloidal stability.

Efficient incorporation of mangosteen pericarp extract into the nanoemulsion was confirmed by the high encapsulation efficiency of approximately 89%. This result is highly driven by the hydrophobic character of α-mangostin, the major bioactive constituent of mangosteen pericarp [35]. Due to its poor aqueous solubility and strong affinity for lipid environments, α-mangostin preferentially partitions into the oil phase during nanoemulsion formation, promoting effective retention within the dispersed droplets [36]. A previous study also demonstrated that α-mangostin can be incorporated into nanoemulsion systems because of its compatibility with the lipid phase and the interfacial stabilization provided by surfactants [37]. Transmission electron microscopy further supported successful encapsulation by revealing predominantly spherical droplets with distinct boundaries and no apparent aggregation. The increase in droplet size observed after incorporation of the extract compared with the blank nanoemulsion suggests that phytochemical constituents were successfully accommodated within the oil phase and interfacial region of the nanoemulsion droplets [38].

#### 3.3.4. pH

**Figure 4:**
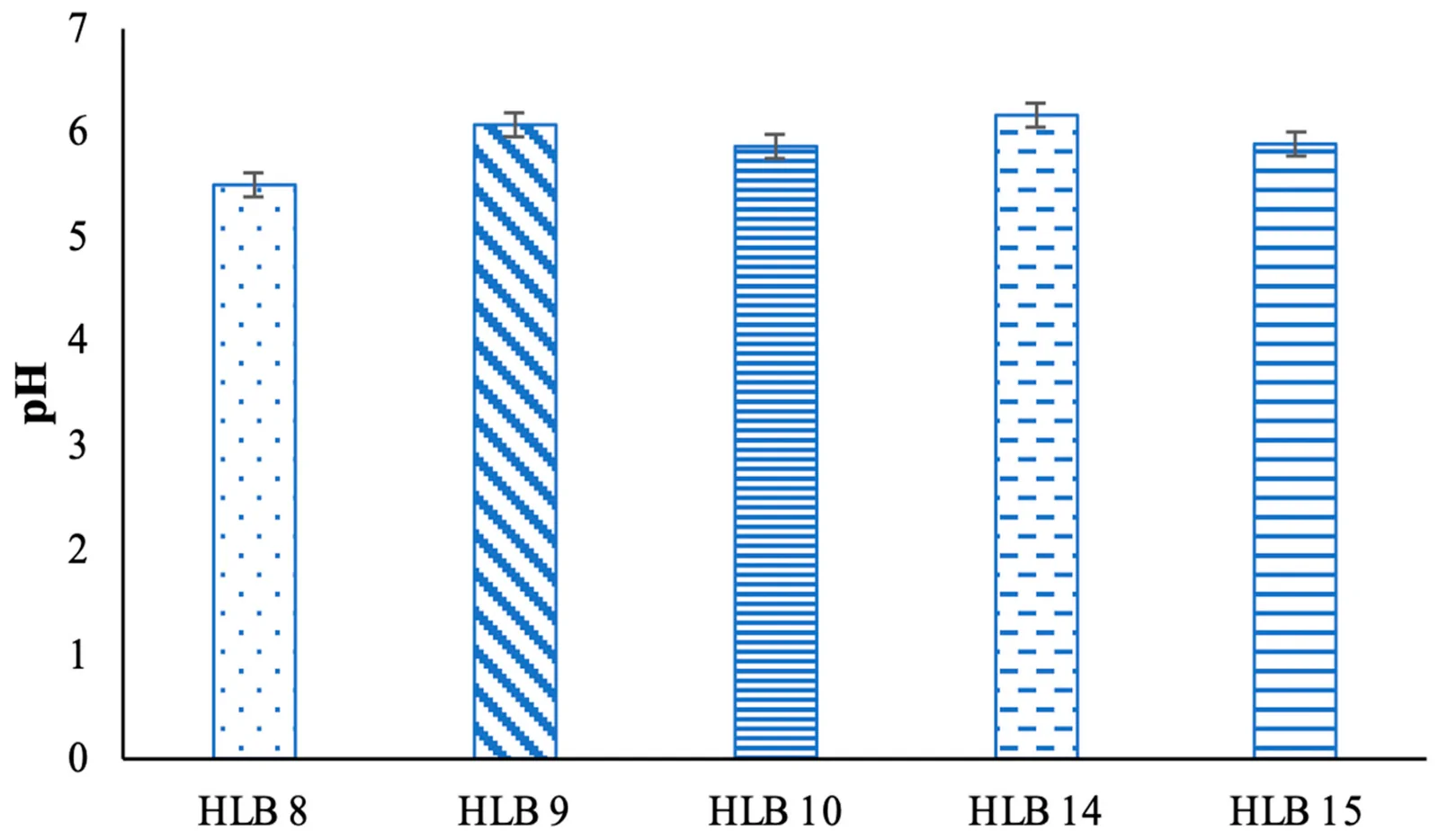
pH values of stable mangosteen pericarp extract nanoemulsions prepared at different HLB values. Data are presented as mean ± SD (n = 3).

The pH values of the stable formulations ranged from 5.51 ± 0.19 to 6.17 ± 0.42 (Figure 4). HLB 8 exhibited the lowest pH value, whereas HLB 14 showed the highest pH value. Statistical analysis revealed no significant difference in pH between the optimized formulation (HLB 10) and the remaining formulations (*p* > 0.05), indicating that variation in surfactant HLB had minimal influence on the acidity of the nanoemulsion system.

#### 3.3.5. Viscosity

**Figure 5:**
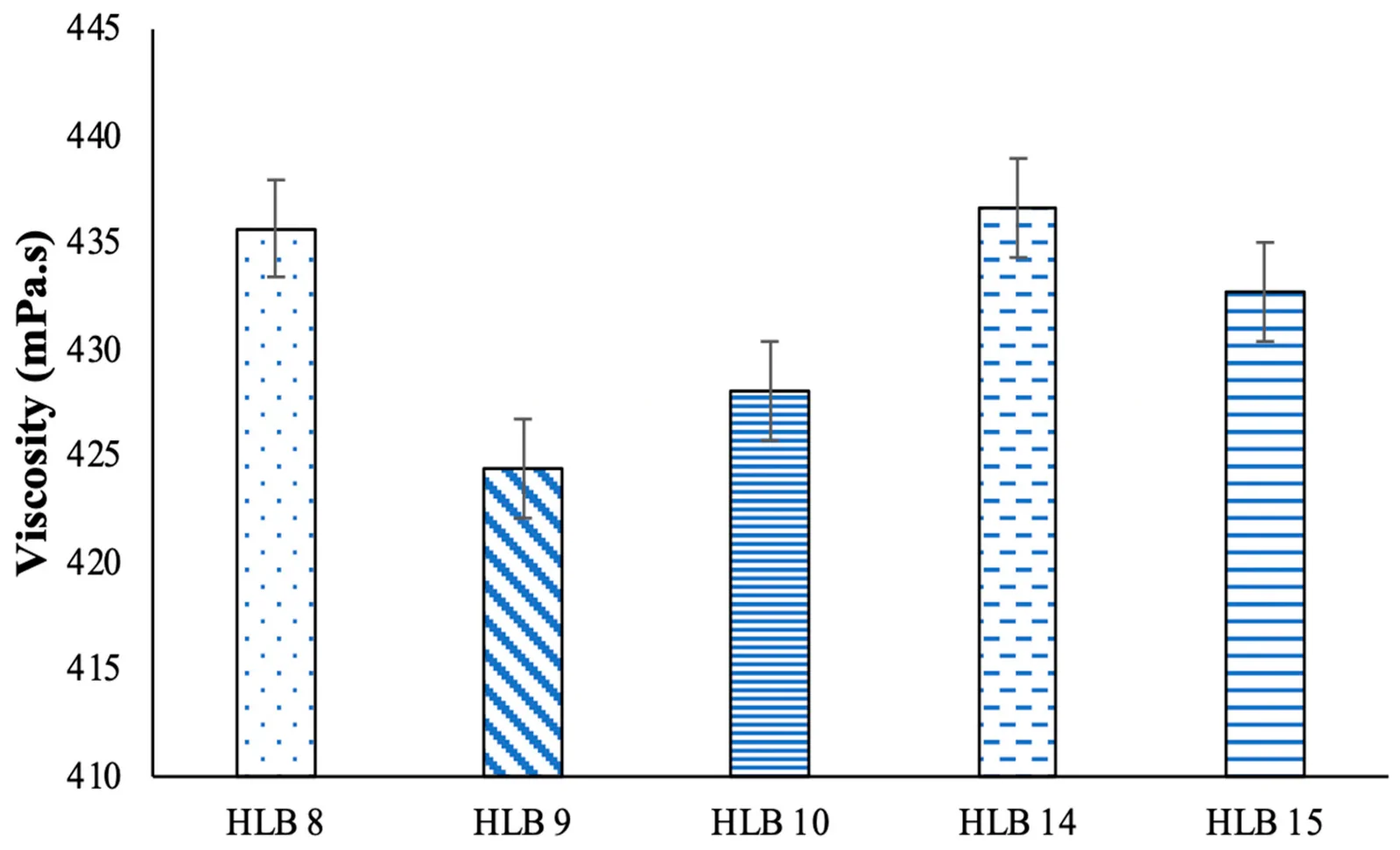
Viscosity of stable mangosteen pericarp extract nanoemulsions prepared at different HLB values. Data are presented as mean ± SD (n = 3).

The viscosity values ranged from 424.40 ± 0.50 to 436.63 ± 1.21 mPa·s (Figure 5). HLB 9 produced the lowest viscosity, while HLB 14 exhibited the highest value. Although statistical comparison using Welch’s *t*-test indicated a significant difference between HLB 10 and the pooled non-optimal formulations (*p* = 0.014), the absolute variation among formulations was small (<3%), suggesting limited practical impact on the rheological behavior of the nanoemulsions.

### 3.4. Morphological Characterization

**Figure 6:**
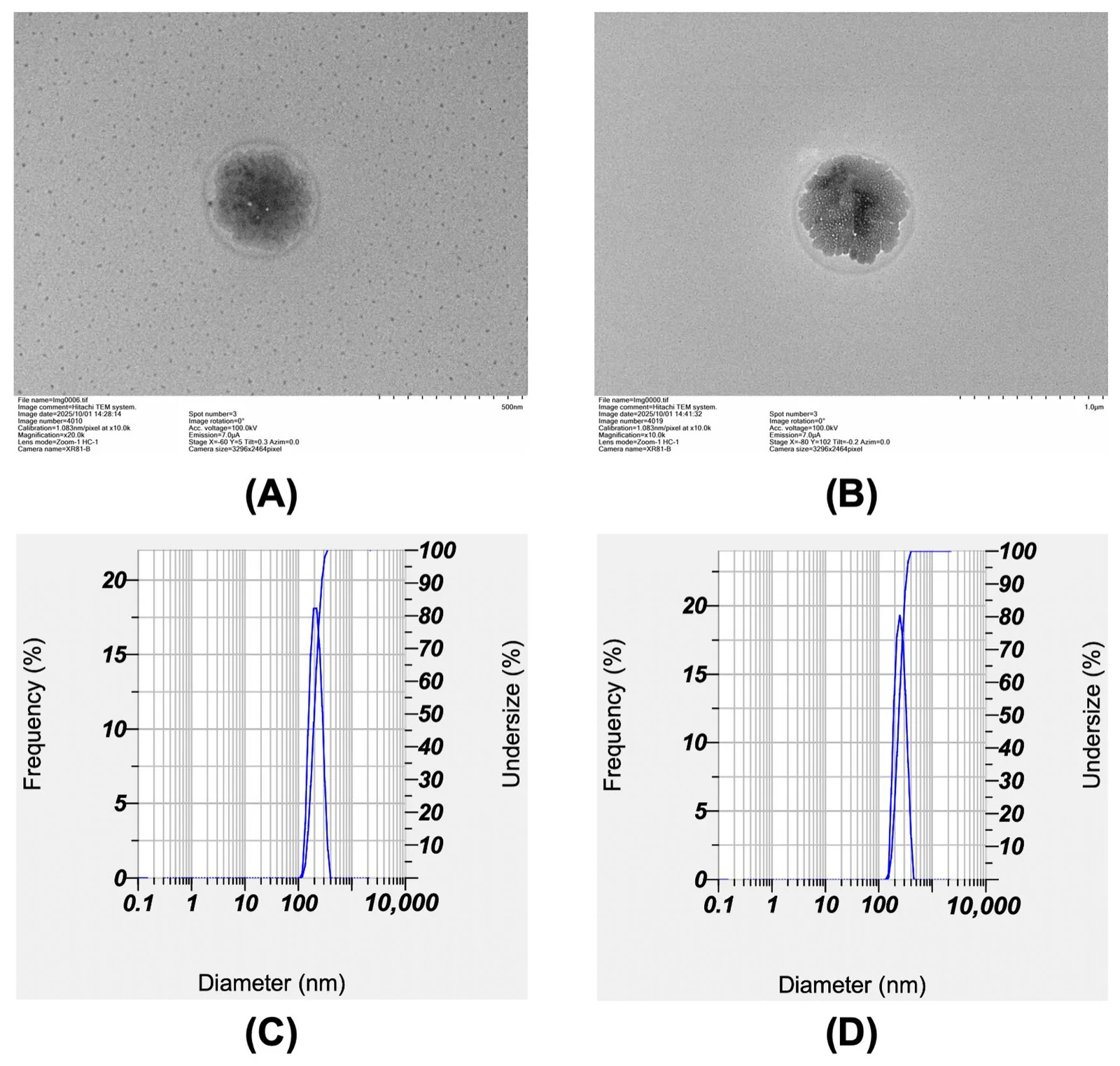
Morphological and particle size characterization of the optimized nanoemulsion system. (**A**) TEM micrograph of blank nanoemulsion. (**B**) TEM micrograph of mangosteen pericarp extract-loaded nanoemulsion. (**C**) Particle size distribution profile of the blank nanoemulsion. (**D**) Particle size distribution profile of the optimized mangosteen pericarp extract nanoemulsion.

The structural morphology of the optimized system was investigated using transmission electron microscopy (TEM). Representative TEM micrographs displaying the blank system alongside the formulation loaded with mangosteen extract are shown in Figure 6A and Figure 6B, respectively. Both formulations exhibited predominantly spherical droplets with distinct boundaries. No evidence of extensive aggregation or coalescence was observed. The nanoemulsion containing mangosteen extract displayed larger droplet dimensions than the blank formulation, consistent with the average droplet diameters determined through dynamic light scattering parameters. Droplet size distribution analysis obtained from PSA measurements (Figure 6C,D) demonstrated a unimodal distribution profile for both formulations. The optimized nanoemulsion maintained a relatively homogeneously narrow dispersion range regardless of the encapsulation of mangosteen extract.

### 3.5. In Vitro Release Behavior

**Figure 7:**
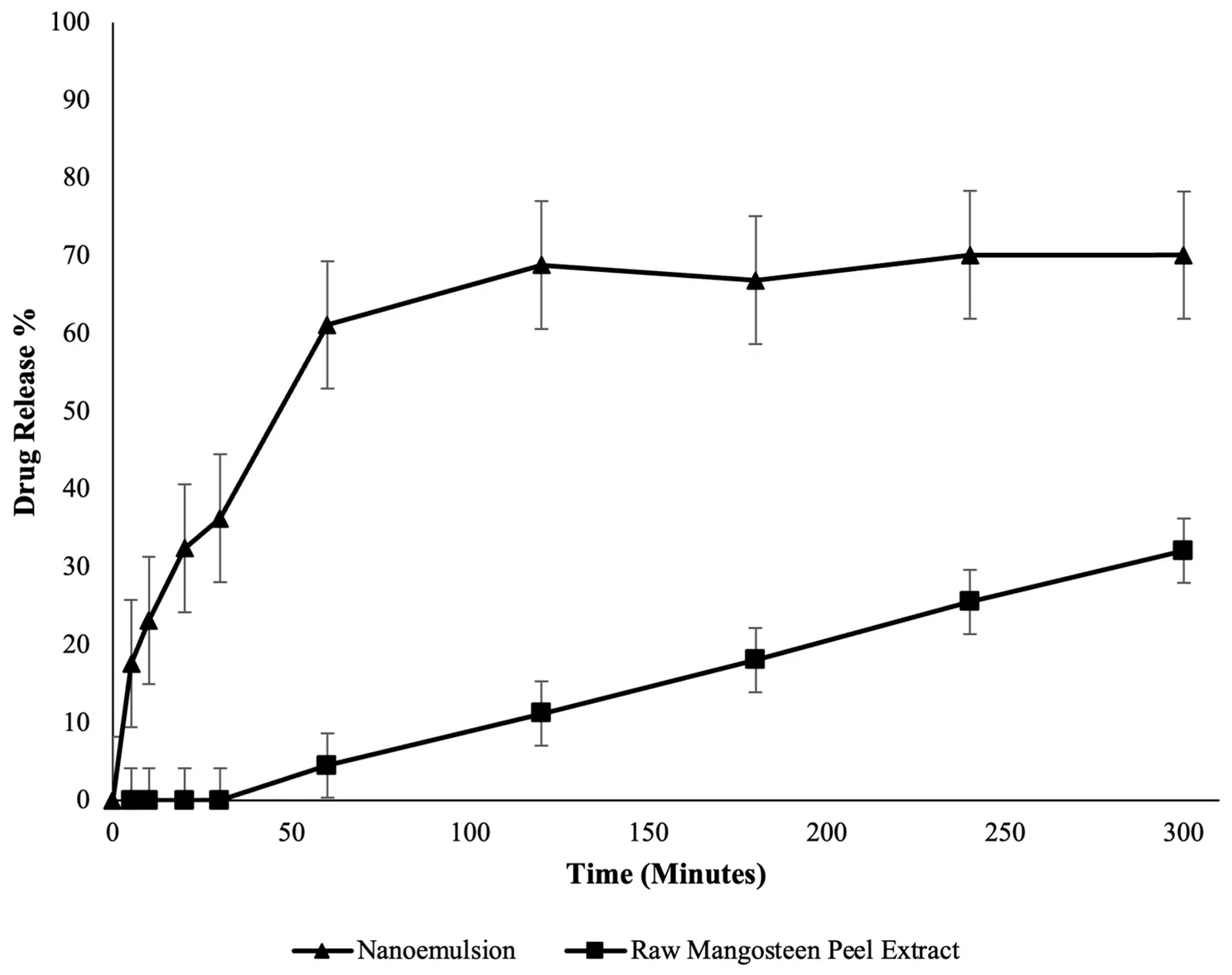
In vitro release profile of mangosteen pericarp extract from nanoemulsion and free extract formulations. Cumulative release (%) was monitored over 300 min under dissolution conditions. Data are expressed as mean ± SD (n = 3).

The release profiles of mangosteen pericarp extract from the nanoemulsion system and free extract are presented in Figure 7. A markedly faster release was observed for the nanoemulsion formulation throughout the study period. At 30 min, the cumulative release of mangosteen extract from the nanoemulsion reached 36.32%, whereas the free extract exhibited only 0.04% release. At 60 min, cumulative release increased to 61.16% for the nanoemulsion and 4.55% for the free extract. This release divergence remained evident during the later stages of the experiment. At 240 min, the nanoemulsion achieved a cumulative release of 70.13%, while the free extract reached 25.54%. Statistical analysis revealed a statistically significant variation between both groups (*p* < 0.05).

Notably, at t = 60 min, the nanoemulsion achieved an approximately 13.4-fold dissolution enhancement, calculated as the ratio of cumulative bioactive release from the nanoemulsion to that of the unformulated extract (%release in nanoemulsion/%release of raw extract = 61.16%/4.55%). This finding is consistent with previous studies showing that lipid-based nanocarriers and nanoemulsions can enhance the dissolution and aqueous dispersion of poorly water-soluble mangosteen bioactives [8,10]. These results support nanoemulsification as an effective strategy to overcome the poor aqueous solubility of mangosteen-derived bioactive compounds. This enhancement became evident during the initial stages of the release study, where the nanoemulsion rapidly released the encapsulated compounds while the free extract remained only minimally dissolved. The improvement can be explained by several complementary mechanisms. First, reduction of droplet dimensions profoundly expands the cumulative boundary surface space that facilitates mass transfer, thereby accelerating dissolution according to the Noyes–Whitney principle [39]. Second, the presence of nonionic surfactants facilitates the solubilization of lipophilic compounds by maintaining them in a dispersed state and reducing the energetic barrier for transfer into the aqueous medium [40]. Third, encapsulation within uniformly distributed oil droplets minimizes aggregation of hydrophobic compounds and enhances their accessibility to the surrounding medium [41]. These findings indicate that the nanoemulsion system effectively overcame one of the major physicochemical limitations associated with α-mangostin-rich extracts.

To investigate the underlying mass transfer behavior, the release profiles were fitted to several mathematical models, such as zero-order, first-order, Higuchi, and Korsmeyer–Peppas equations (Table 4).

Among the evaluated mathematical models, the Korsmeyer–Peppas equation provided the peak value for the determination coefficient (R^2^ = 0.9382), signifying that the mass transfer kinetics followed a simultaneous blending of molecular diffusion and polymeric chain relaxation processes [42]. The calculated diffusion exponent (n = 0.48) points to an anomalous (non-Fickian) transport behavior, a phenomenon commonly observed in nanostructured delivery systems where multiple transport pathways simultaneously contribute to drug release [43]. Such behavior indicates that the discharge kinetics do not depend exclusively upon passive diffusion but are significantly regulated by dynamic interactions occurring within the dispersed nanoemulsion structure.

### 3.6. Radical Scavenging Properties

**Figure 8:**
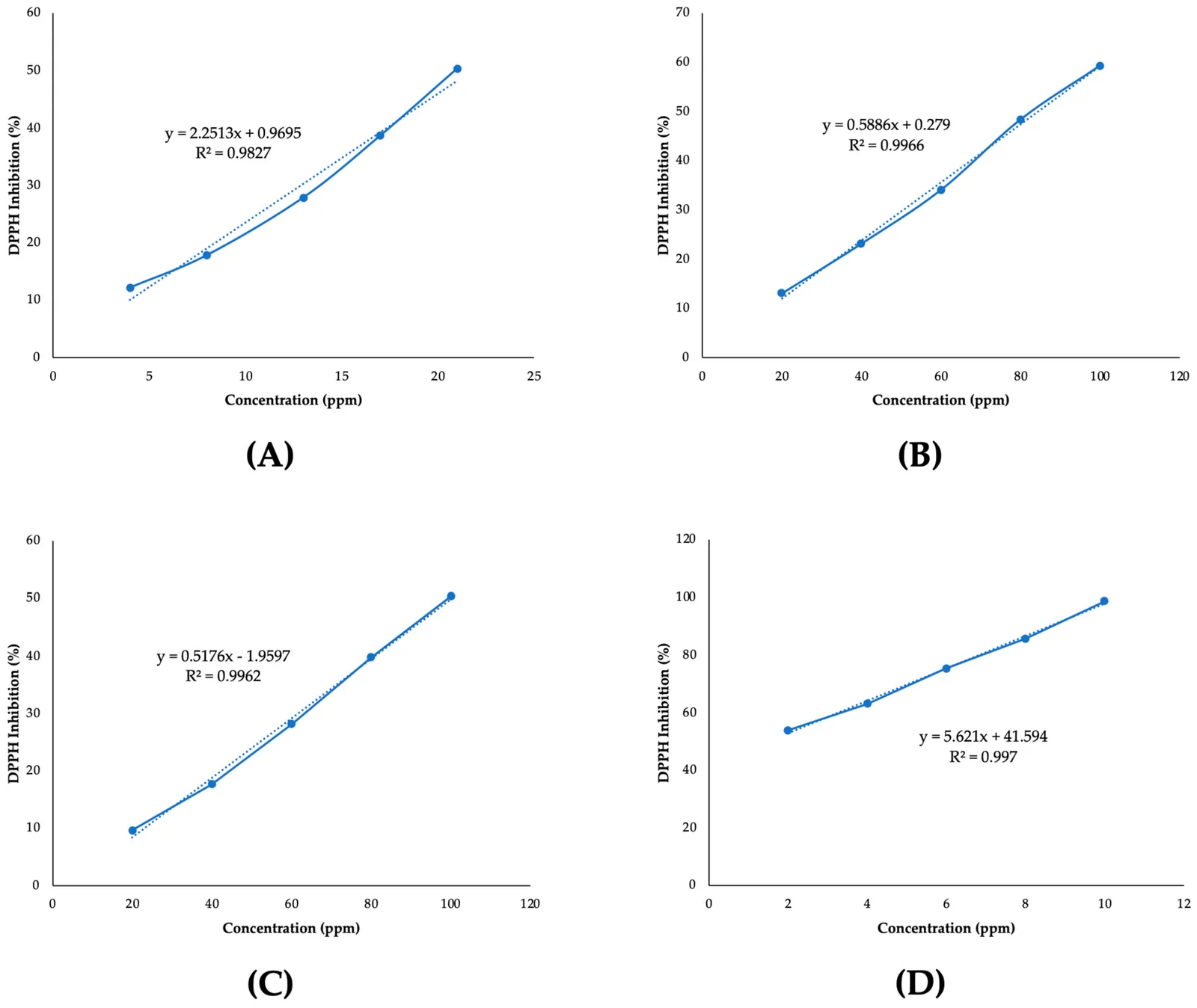
Concentration-dependent DPPH radical scavenging activity and IC_50_ determinations. Percentage of DPPH radical inhibition plotted against concentration (μg/mL) for (**A**) raw mangosteen pericarp extract, (**B**) optimized mangosteen pericarp extract nanoemulsion, (**C**) sacha inchi oil, and (**D**) ascorbic acid (reference standard). Half-maximal inhibitory concentration (IC_50_) values were extrapolated from the concentration–response curves.

**Figure 9:**
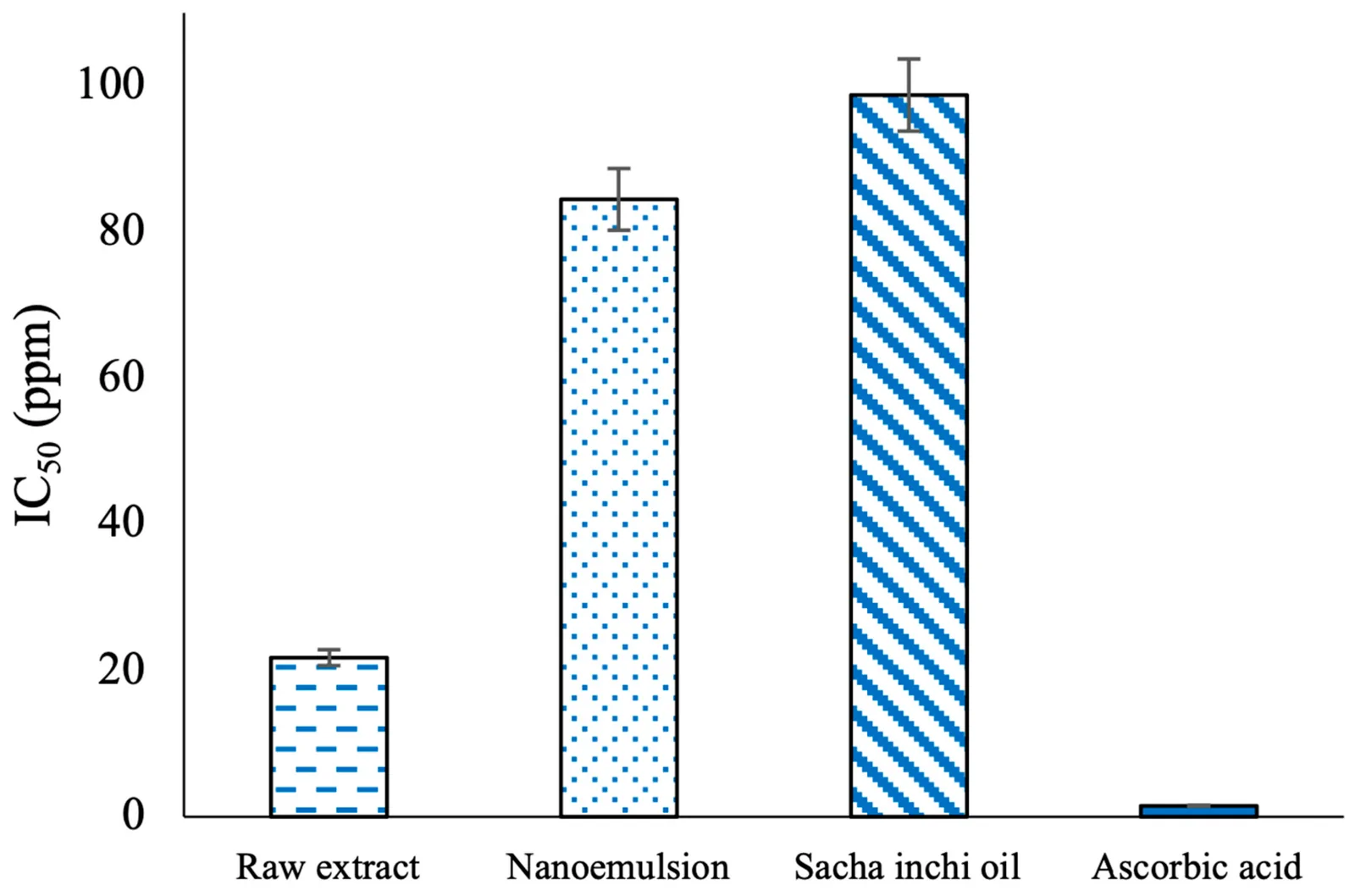
Antioxidant activity of mangosteen pericarp extract, sacha inchi oil, and optimized nanoemulsion determined using the DPPH radical scavenging assay. IC_50_ values were calculated from concentration–response curves and compared with ascorbic acid as the reference antioxidant.

The radical scavenging capacity of the mangosteen pericarp sample, sacha inchi oil, and the optimized nanoemulsion formulation was measured via the DPPH spectrophotometric method. The computed IC_50_ metrics are depicted in Figure 8 and Figure 9. Mangosteen pericarp extract exhibited the strongest antioxidant activity among the tested samples, with an IC_50_ value of 21.68 ppm. Following incorporation into the nanoemulsion system, the antioxidant activity remained substantial, yielding an IC_50_ value of 84.48 ppm. Sacha inchi oil demonstrated an IC_50_ value of 98.76 ppm. A strong linear relationship between concentration and radical scavenging activity was observed for all samples, as indicated by coefficients of determination (R^2^) exceeding 0.99. The antioxidant performance of all tested mixtures was inferior compared to the positive control, ascorbic acid (IC_50_ = 4.40 ppm).

The antioxidant activity assessment revealed that mangosteen pericarp extract possessed very strong radical-scavenging activity, whereas the nanoemulsion maintained strong antioxidant activity following encapsulation. Although the IC_50_ value increased after incorporation into the nanoemulsion system, this observation does not necessarily indicate a loss of functional potential. Several studies have reported similar findings for encapsulated antioxidant compounds, where the carrier matrix partially limits direct contact between the bioactive compound and DPPH radicals during in vitro analysis [29]. In such systems, a fraction of the antioxidant molecules remains associated with the internal phase and therefore becomes less immediately accessible during the assay. Consequently, the measured antioxidant activity may underestimate the actual antioxidant reservoir retained within the nanocarrier. Considering the marked improvement in dissolution behavior and encapsulation efficiency observed in the present study, the reduction in apparent antioxidant activity may represent a trade-off associated with successful incorporation of α-mangostin into the nanoemulsion structure [8].

### 3.7. Evaluation of Formulation Stability

The physical and chemical integrity of the optimized mangosteen pericarp extract nanoemulsion was evaluated by exposing the formulations to long-term storage parameters (25 ± 2 °C accompanied by 75 ± 5% RH) alongside stress-induced accelerated environments (40 ± 2 °C accompanied by 75 ± 5% RH). Changes affecting the mean droplet diameter, polydispersity values, zeta potential, viscosity, pH levels, antioxidant activity, as well as α-mangostin content were monitored over one month of storage (Table 5).

The optimized nanoemulsion exhibited satisfactory colloidal characteristics at the initial time point, with a mean droplet size of 490.03 ± 32.12 nm, a PDI of 0.66 ± 0.01, and a net surface charge of −52.73 ± 0.09 mV. After one month of storage, the formulation experienced negligible fluctuations in its average droplet diameter under each of the tested storage conditions, indicating that the nanoemulsion maintained its dispersed structure throughout the study period. The zeta potential remained highly negative after storage, with values ranging from −44.75 to −51.85 mV, suggesting that sufficient electrostatic repulsion was retained to prevent extensive droplet aggregation. The relatively stable colloidal indicator values suggest the lipidic nano-matrix successfully maintained its colloidal integrity during storage, demonstrating adequate physical stability of the dispersed system [44]. Similarly, pH values remained within a narrow range throughout storage, indicating good physicochemical compatibility of the formulation.

In contrast, antioxidant activity and α-mangostin content showed noticeable reductions during storage. The IC_50_ value increased from 84.48 ppm to 101.05 ppm and 136.66 ppm under long-term and accelerated storage conditions, respectively, indicating a decline in radical scavenging activity. Likewise, α-mangostin content decreased from 36.71% to 15.39% and 17.04% after storage. The decrease in α-mangostin concentration suggests that degradation of bioactive constituents occurred even though the nanoemulsion structure remained largely intact. Since α-mangostin is recognized as one of the principal antioxidant compounds in mangosteen pericarp extract, its degradation is likely responsible for the reduced radical-scavenging capacity observed after storage [45].

Interestingly, storage at 40 °C failed to induce marked fluctuations in particle size as well as zeta potential compared with storage at 25 °C, further supporting the robustness of the colloidal system. However, the higher IC_50_ value obtained under accelerated conditions indicates that elevated temperature accelerated chemical degradation processes. This observation aligns with prior literature documenting the vulnerability of xanthones and other polyphenolic constituents toward oxidative pathways, thermal breaking, and isomeric transformation throughout the aging period [46]. Therefore, the primary stability challenge of the developed formulation appears to be preservation of the encapsulated phytochemicals rather than maintenance of the nanoemulsion structure itself.

Regarding long-term stability, a clear distinction must be made between physical and chemical stability. While the high zeta potential (>50 mV) effectively prevented severe phase separation and droplet coalescence (maintaining physical integrity), the gradual reduction in α-mangostin content and antioxidant efficacy during storage is primarily governed by chemical degradation pathways. α-Mangostin is susceptible to oxidative degradation upon exposure to dissolved oxygen, light, and ambient temperature [47]. Furthermore, sacha inchi oil’s high content of polyunsaturated fatty acids (such as α-linolenic acid) renders the lipid core prone to lipid peroxidation, which generates free radicals that can accelerate the chemical oxidation of encapsulated polyphenols [48].

To mitigate the limited chemical stability of α-mangostin during long-term storage, future formulation strategies should incorporate lipophilic secondary antioxidants (such as α-tocopherol) directly into the sacha inchi oil phase to terminate lipid peroxidation chain reactions [49]. Furthermore, protecting the final nanoemulsion from light exposure using amber glass packaging, flushing with inert nitrogen gas (N_2_), and maintaining cold storage conditions (4 °C) are recommended strategies to preserve the chemical integrity of the active constituents.

Overall, the results demonstrate that nanoemulsification using sacha inchi oil represents an encouraging approach to optimize the administration of mangosteen pericarp extract. The newly developed system achieved high encapsulation efficiency, favorable physicochemical characteristics, substantial enhancement of dissolution behavior, and satisfactory colloidal stability during storage. This study has several limitations that should be acknowledged. First, the stability evaluation was conducted for only one month; therefore, the long-term physical and chemical stability of the formulation could not be determined. The decrease in α-mangostin content and antioxidant activity during storage indicates the need for further long-term stability studies. Second, the present study was limited to in vitro physicochemical characterization, antioxidant activity, and dissolution testing. Therefore, the potential improvement in α-mangostin bioavailability could not be confirmed. Further in vivo pharmacokinetic and bioavailability studies are needed to determine whether the improved dissolution of the nanoemulsion can translate into increased systemic availability of α-mangostin.

## 4. Conclusions

In conclusion, a sacha inchi oil-based nanoemulsion carrying mangosteen pericarp extract was successfully fabricated using high-shear homogenization followed by ultrasonication. The optimized formulation (HLB 10) yielded high encapsulation efficiency (89.14 ± 0.19%) and robust electrostatic repulsion (zeta potential of −52.73 ± 0.09 mV), despite presenting a relatively large submicron droplet size (490.03 ± 32.12 nm) and moderate polydispersity. The nanoemulsion successfully overcame the solubility barrier of the extract, achieving a 13.4-fold enhancement in apparent in vitro dissolution compared to the unformulated raw powder. While the formulation retained functional antioxidant potential, a reduction in radical scavenging activity (IC_50_ = 84.48 ppm vs. 21.68 ppm for raw extract) was observed due to carrier entrapment. Furthermore, the marked loss of α-mangostin content over 1 month indicates that chemical stability remains a critical limitation of this lipid matrix. Overall, while sacha inchi oil serves as an effective carrier to enhance extract dissolution, future work must incorporate secondary chemical stabilizers or protective conditions before advancing to in vivo bioavailability and safety evaluations.

## Figures and Tables

**Table 1:** Composition of mangosteen pericarp extract nanoemulsions formulated with different hydrophilic–lipophilic balance (HLB) values of Tween 80/Span 80 surfactant mixtures.

Component (% *w*/*w*)	HLB 8	HLB 9	HLB 10	HLB 11	HLB 12	HLB 13	HLB 14	HLB 15
Mangosteen pericarp extract	1.2	1.2	1.2	1.2	1.2	1.2	1.2	1.2
Sacha inchi oil	30.0	30.0	30.0	30.0	30.0	30.0	30.0	30.0
Span 80	6.54	5.61	4.67	3.74	2.80	1.86	0.92	0.00
Tween 80	3.46	4.39	5.33	6.26	7.20	8.14	9.08	10.00
BHT	0.05	0.05	0.05	0.05	0.05	0.05	0.05	0.05
Sodium benzoate	0.10	0.10	0.10	0.10	0.10	0.10	0.10	0.10
Xanthan gum	0.30	0.30	0.30	0.30	0.30	0.30	0.30	0.30
Purified water	add to 100	add to 100	add to 100	add to 100	add to 100	add to 100	add to 100	add to 100

**Table 2:** Physicochemical indicators (droplet size, PDI, zeta potential, and encapsulation efficiency) of mangosteen pericarp extract nanoemulsions.

Formulation	Droplet Size (nm)	PDI	Zeta Potential (mV)	EE (%)
HLB 8	974.30 ± 85.84	0.89 ± 0.03	−54.01 ± 1.19	84.76 ± 4.48
HLB 9	987.27 ± 67.49	0.82 ± 0.01	−56.83 ± 0.05	89.06 ± 0.01
HLB 10	490.03 ± 32.12	0.66 ± 0.01	−52.73 ± 0.09	89.14 ± 0.19
HLB 14	880.07 ± 6.54	0.68 ± 0.05	−60.70 ± 1.13	87.82 ± 0.04
HLB 15	605.73 ± 35.79	0.65 ± 0.04	−57.51 ± 3.58	89.04 ± 0.29

**Table 3:** Physicochemical characteristics of optimized nanoemulsions.

Formulation	Particle Size (nm)	PDI	Zeta Potential (mV)
Blank nanoemulsion	191.03 ± 2.76	0.35 ± 0.01	−42.50 ± 0.26
Mangosteen extract nanoemulsion	490.03 ± 32.12	0.66 ± 0.01	−52.73 ± 0.09

**Table 4:** Release kinetic modeling of the optimized nanoemulsion.

Model	R^2^
Zero-order	0.6952
First-order	0.7458
Higuchi	0.8451
Korsmeyer–Peppas	0.9382

**Table 5:** Stability parameters of the optimized nanoemulsion during storage.

Time (Month)	Storage Condition	Particle Size (nm)	PDI	Zeta Potential (mV)	Viscosity (mPa·s)	pH	IC_50_ (ppm)	α-Mangostin (%)
0	Initial	490.03	0.660	−52.73	428.03	5.88	84.48	36.71
1	25 ± 2 °C/75 ± 5% RH	476.70	0.680	−44.75	369.67	6.33	101.05	15.39
	40 ± 2 °C/75 ± 5% RH	489.65	0.410	−51.85	398.67	6.11	136.66	17.04

## Data Availability

Data will be made available on request.
